# Education and Health

**Published:** 1872-12

**Authors:** 


### HALL'S

### JOURNAL OF HEALTH.

Vol. XIX. — DECEMBER, 1872. — No. XII.

### EDUCATION AND HEALTH.

The educated live longer than the illiterate; the rich, longer than the poor; the good, longer than the bad. Education tends to religion; religion promotes thrift, which gives the means of convenience, comfort, and health. Especially does the education of the mother tend to elevate the race in well-doing, piety, and robustness of constitution, by means of a temperate, industrious, and busy life, all of which are the natural outgrowths of sterling religious principle. Hence, the proper education of our daughters is a subject of momentous interest. One of the best educational institutions for girls in the United States is Vassar College, Poughkeepsie, N. Y., which commenced operations in 1865, and seven years later had upwards of four hundred students.

The whole expense of tuition, forty weeks' board, furnished rooms, fuel, lights, servants, attendance, and washing, is four hundred dollars; those who need aid have it afforded, to the extent of two hundred dollars a year, if a high degree of scholarship is attained, as a reward for proficiency, not as a charity. The session begins September 18, and ends June 26. The regular college course is four years. At the end of two years the pupil has choice of studies. To enter college proper, a searching examination has to be passed. As the institution is always full, applications for vacancies are considered in their turn; hence, parents desiring to send their daughters can apply at any time, and in due season their turn will come. ' Address John H. Raymond, LL. D., President, Poughkeepsie, Dutchess County, N. Y. The college is open to all denominations of Christians, but is subject to none.

The founder, Matthew Vassar, was a man of large wealth, which his wife helped to accumulate, by her industry, economy, and wise counsels. The editor has heard native residents of the town say, that Mrs. Vassar was very illiterate; so much so, that when gentlemen called on the husband, he preferred to dine them at the hotel, to avoid the exposure of her want of culture. This excellent woman, in very early life, had her own parents' orphaned children left to her care, compelling her to forego all advantages of a school education. She completed her task, nobly performed her duty, and when these younger children were off her hands, she married Mr. Vassar.

It is reasonable to conclude that the circumstances involved in this narration were the germs which fructified in Matthew Vassar's brain and heart, bringing forth eventually as the product, Vassar College, one of the very noblest educational institutions for women in any part of the globe. Many a reader will be glad to learn that Maria, the daughter of the lamented Professor Mitchell, the most eloquent astronomical lecturer of any age, is the able conductor of the astronomical observatory.

In this connection, it is pertinent to suggest, in such a utilitarian age as this, that the education of a girl should never be considered as complete, or even half completed, until she thoroughly studies and comprehends the science of bread-making, and has become a thorough adept in the practice of the art.

She should be able to cut and fit and make her own dresses.

She should know how to keep a daily account of all the money received by her, from what sources, and its outlay.

She should know how to select the best meats and vegetables and fruits of all kinds, how to prepare them in the best manner, and how to make them go the farthest.

She should understand the science and art of taking the best possible care of her clothing, so as to make it always appear new and clean, until it gets old and worn by fair usage; and then be able to change and turn and alter so as to make it appear almost as good as new, whether dresses or bonnets.

As every true woman calculates on getting married sooner or later, she should learn beforehand the art of keeping her husband, rather than that of getting one; how to keep a husband's respect and love, rather than manœuvre how to obtain them; to this end she should sedulously cultivate purity of character, self-respect, control of temper, equanimity, integrity, truthfulness in all things. Wives that are to be, should be taught that husbands require coöperation, sympathy, encouragement; that they love praise, the more so as they affect to deny it; that they love order, quiet, and a tidy table, although there be nothing upon it but a loaf and a potato.

It would be an admirable thing, if, at the age of twelve years, when Mrs. Vassar, without previous preparation, took charge of a motherless family of children, mothers should give an only daughter instruction, or if several, "turns," in "setting the table," tidying up their own rooms, darning the stockings of the family, sorting out the clothing, mending rents, sewing on buttons, making tea and coffee and cake; then in making and baking a loaf of bread, and, by degrees, how to broil a steak or a chicken, how to conduct a roast; taking them to market occasionally, and showing them how to select the best of everything. Almost any little girl will not only enter upon these instructions willingly, but will take an intense delight and a commendable pride in carrying them out. All these things can be done by the mother without any more detraction of time from school studies, than health requires; and even if it took half of every day, and double the time for schooling, the general health will be better in the end, while the girl will have been educated to her wifely duties, and will make a

HELPMEET

for the man who makes her the choice of his life.

That mother teaches her daughter ignobly, who brings her up to suppose that it is the husband's duty to support the family. Husband and wife should work into each other's hands. While it is the duty of the man to get, it is the duty of the woman to take care of what is gotten ; the woman who fails of this is no true wife, and does not deserve a good husband.

The study of economies could be made intensely interesting to young girls ; take for example

THE POTATO.

Take it in hand, and point out the "eyes ;" give the information that if the potato is cut into as many pieces as there are "eyes," each will grow and have at the roots several potatoes; and that a few bushels of potatoes, planted in an acre of land, have been known to yield over three hundred bushels in return, thus paying man handsomely for his labor; that persons can almost live on potatoes; that they constitute the main food of many families; that while good for food for man and beast, starch is made out of them to stiffen dresses and otherwise aid in beautifying the clothing. Then instructions might be given as to the simplest and best and most economical methods of preparation for the table, giving the child the opportunity of cooking them in various ways herself; and encourage and praise her up so as to bring out all her force in doing the thing better and better, until she approaches perfection.

For the mother's instruction the following statements are made: —

It may be safe to say that not more than one family in fifty knows how to cook a potato so as to make it most luscious and go the farthest.

There is a very thin outer skin on the potato, thinner than thin letter-paper, thinner than a wafer; it is, perhaps, not thicker than the twentieth part of an inch.

Immediately under this skin is the best part of the vegetable, the part which makes flesh and gives strength; this part is not more than the tenth of an inch thick; all below that, all the remainder of the potato, is destitute of nourishment; it only warms; it gives no strength, it is mere starch. Hence, in peeling a potato, the most valuable part of it is thrown to the hogs or other domestic animals, and the least valuable part is put on the table. The best way to cook a potato is to do so with the peeling on it, and take it off with the finger-nails or with a cloth, after it is cooked; then only the skin is removed, without any portion of the real nutriment adhering to it. It is very wasteful to roast or bake potatoes; they should either be boiled with their skins or "jackets" on, or should be steamed; in fact, they should never be cooked in any other way; for thus, not only is all the nutriment saved, but it is prepared in the most palatable and digestible manner possible. Wash the potatoes thoroughly and quickly in cold water, put them at once in an iron saucepan with a tight lid, and put it over the fire, as if to boil them, without any water whatever, for they are already wet, and the moisture inside the potatoes amounts to three fourths of the weight of the whole, thus affording steam enough to cook them, and sooner than boiling water would do it; for that is only two hundred and twelve degrees hot, while steam is three or four hundred and more; this method of cooking makes the potatoes dry, rich, and tasteful, without burning, and they come on the table so delightfully mealy and sweet and nutritious, that you will wonder that they should be cooked in any other way, ever more. The vessel containing the potatoes should not be more than two thirds full, so as to leave room for the steam to do its work. Until you can tell pretty well how long it will take to cook, the lid may be lifted to stick a fork in, and replaced quickly. It is better also to keep the vessel shaken back and forth, to prevent burning. As soon as they are turned out, take off the outer skin, and place them on the table. Or they can be mashed, adding a little milk.

Another excellent method is to slice the potatoes very thin, taking the outer skin off first; then place them in a saucepan as before, shaking them all the time; in ten minutes they are ready for the table.

It is pitiful to think on how many tables, year in and year out, potatoes come sodden, hard, and many with all the best part peeled off.

Let the thoughtful mother begin right away, and give herself no rest until she has taught every daughter she has, to do most thoroughly and well the following things:—

To make a cup of coffee.

To draw a dish of tea.

To bake a loaf of bread.

To cook a potato.

To broil a steak or chicken.

To cut, fit, and make a dress.

To set a tidy table.

To have a wife of these seven abilities, or to know how to make her servants competent to them, would give many a millionaire more real pleasure than the addition of tens of thousands to his fortune; would extort more respect and affection than if she were the most beautiful of women. Neither blood nor beauty are capable of infusing domestic happiness into a family, half as much as an ability to bring to it material comfort, and the gratification of the tastes and appetites which were intended to be satisfied, as a means of filling up the cup of life's pleasures; and to do which is, to a certain extent, the duty of all; for we are put here to enjoy as well as to labor, each being equally a duty to ourselves and to others.

